# Effect of rivastigmine as an adjunct to usual care with haloperidol on duration of delirium and mortality in critically ill patients: a multicentre, double-blind, placebo-controlled randomised trial

**DOI:** 10.1186/cc9759

**Published:** 2011-03-11

**Authors:** MM Van Eijk, KC Roes, ML Honing, MA Kuiper, A Karakus, M Van der JAgt, PE Spronk, WA Van Gool, RC Van der Mast, J Kesecioglu, AJ Slooter

**Affiliations:** 1 University Medical Center Utrecht, the Netherlands; 2 Medical Center Alkmaar, the Netherlands; 3 Medical Center Leeuwarden, the Netherlands; 4 Diakonessenhuis Utrecht, the Netherlands; 5 Erasmus Medical Center, Rotterdam, the Netherlands; 6 Gelre Hospitals, Apeldoorn, the Netherlands; 7 Academic Medical Center, Amsterdam, the Netherlands; 8 Leiden University Medical Center, Leiden, the Netherlands

## Introduction

Delirium is frequently diagnosed in critically ill patients and is associated with adverse outcome. Impaired cholinergic neurotransmission seems to have an important role in the development of delirium. We aimed to establish the effect of the cholinesterase inhibitor rivastigmine on the duration of delirium in critically ill patients.

## Methods

Patients (aged ≥18 years) who were diagnosed with delirium were enrolled from six ICUs in the Netherlands, and treated between November 2008 and January 2010. Patients were randomised (1:1 ratio) to receive an increasing dose of rivastigmine or placebo, starting at 0.75 ml (1.5 mg rivastigmine) twice daily and increasing in increments to 3 ml (6 mg rivastigmine) twice daily from day 10 onwards, as an adjunct to usual care based on haloperidol. The trial pharmacist generated the randomisation sequence by computer, and consecutively numbered bottles of the study drug according to this sequence to conceal allocation. The primary outcome was the duration of delirium during hospital admission. Analysis was by intention to treat. Duration of delirium was censored for patients who died or were discharged from hospital while delirious. Patients, medical staff, and investigators were masked to treatment allocation. Members of the data safety and monitoring board (DSMB) were unmasked and did interim analyses every 3 months.

## Results

Although a sample size of 440 patients was planned, after inclusion of 104 patients with delirium who were eligible for the intention-to-treat analysis (*n *= 54 on rivastigmine, *n *= 50 on placebo), the DSMB recommended that the trial be halted because mortality in the rivastigmine group (*n *= 12, 22%) was higher than in the placebo group (*n *= 4, 8%; *P *= 0.07). Median duration of delirium was longer in the rivastigmine group (5.0 days, IQR 2.7 to 14.2) than in the placebo group (3.0 days, IQR 1.0 to 9.3; *P *= 0.06).

## Conclusions

Rivastigmine did not decrease duration of delirium and might have increased mortality so we do not recommend use of rivastigmine to treat delirium in critically ill patients.

